# Adhesion and Migration Response to Radiation Therapy of Mammary Epithelial and Adenocarcinoma Cells Interacting with Different Stiffness Substrates

**DOI:** 10.3390/cancers12051170

**Published:** 2020-05-06

**Authors:** Valeria Panzetta, Giuseppe La Verde, Mariagabriella Pugliese, Valeria Artiola, Cecilia Arrichiello, Paolo Muto, Marco La Commara, Paolo A. Netti, Sabato Fusco

**Affiliations:** 1 Centro di Ricerca Interdipartimentale sui Biomateriali, Università degli Studi di Napoli Federico II, Piazzale Tecchio 80, 80125 Napoli, Italy; nettipa@unina.it; 2 Centre for Advanced Biomaterial for Health Care, Istituto Italiano di Tecnologia, Largo Barsanti e Matteucci 53, 80125 Napoli, Italy; 3 Istituto Nazionale di Fisica Nucleare, INFN sezione di Napoli, Via Cinthia ed. 6, 80126 Napoli, Italy; glaverde@na.infn.it (G.L.V.); pugliese@na.infn.it (M.P.); marco.lacommara@unina.it (M.L.C.); 4 Dipartimento di Farmacia, Università degli Studi di Napoli Federico II, Via Montesano 49, 80131 Napoli, Italy; 5 Dipartimento di Fisica “Ettore Pancini”, Università degli Studi di Napoli Federico II, Via Cinthia ed. 6, 80126 Napoli, Italy; val.artiola@gmail.com; 6 Radiotherapy Unit, Istituto Nazionale Tumori-IRCCS-Fondazione “G. Pascale”, Via Semmola, 53, 80131 Naples, Italy; c.arrichiello@istitutotumori.na.it (C.A.); p.muto@istitutotumori.na.it (P.M.)

**Keywords:** breast cancer, mechanobiology, cell motility, extracellular matrix stiffness, radiotherapy

## Abstract

The structural and mechanical properties of the microenvironmental context have a profound impact on cancer cell motility, tumor invasion, and metastasis formation. In fact, cells react to their mechanical environment modulating their adhesion, cytoskeleton organization, changes of shape, and, consequently, the dynamics of their motility. In order to elucidate the role of extracellular matrix stiffness as a driving force in cancer cell motility/invasion and the effects of ionizing radiations on these processes, we evaluated adhesion and migration as biophysical properties of two different mammary cell lines, over a range of pathophysiological stiffness (1–13 kPa) in a control condition and after the exposure to two different X-ray doses (2 and 10 Gy, photon beams). We concluded that the microenvironment mimicking the normal mechanics of healthy tissue has a radioprotective role on both cell lines, preventing cell motility and invasion. Supraphysiological extracellular matrix stiffness promoted tumor cell motility instead, but also had a normalizing effect on the response to radiation of tumor cells, lowering their migratory capability. This work lays the foundation for exploiting the extracellular matrix-mediated mechanism underlying the response of healthy and tumor cells to radiation treatments and opens new frontiers in the diagnostic and therapeutic use of radiotherapy.

## 1. Introduction

Breast cancer is the most common malignancy and the first leading cause of cancer-related death in European women [1]. In particular, women in perimenopausal and postmenopausal age have a higher risk of getting breast cancer [2], and extensive attempts should be made in order to contain breast cancer incidence and mortality. In this context, radiation therapy (RT) is used as adjuvant therapy to prevent tumor recurrence after breast-conserving lumpectomy and mastectomy. RT can induce dramatic consequence for the cells, by stimulating the production of radical and reactive oxygen species that damage the DNA of cancer cells, leading to the formation of lethal chromosome aberrations (double-stranded breaks and/or lesions) and, consequently, killing them or slowing their growth, as widely reported in the literature [3,4,5,6]. In any case, the mechanism of the response of the cell cytoskeleton to RT in relationship to the mechanical microenvironment in which cell resides has not yet been elucidated in a comprehensive way. Nowadays, it is widely recognized that cell cytoskeleton and extracellular matrix (ECM) have key roles in the maintenance of the correct functioning of many tissue processes that, if altered, have a determinant contribution in cancer progression. In fact, it has been shown that tumor cells have a less organized and structured cytoskeleton with lower cell mechanical and cyto-adhesive properties compared to their healthy counterparts. Furthermore, the dynamic alteration of the actin cytoskeleton has strong implications on motility, invasion, and metastatic potential of cancer cells [7]. On the other hand, changes in ECM composition and architecture result in a stiffening process of the matrix that activates cell proliferation and a consequent invasion mechanism [8,9,10]. Recently, several research groups have started to focus their attention on the study of possible impacts of radiation on the cell cytoskeleton and its associated functions. In particular, it has been extensively investigated the way ionizing radiations can influence motility, a prerequisite for the formation of metastasis, and for the invasiveness itself of surviving cancer cells both in vitro and in pre-clinical/clinical experimental studies. The observations made in vitro experiments indicate that the effects of radiation on cell motility depend strongly on the specific cell phenotype and the dose delivered to the cells. So far, it has been reported that irradiation has very different impacts on the motility of four different glioblastoma cell lines, inducing a very strong inhibition of in vitro invasion only on one of them (A-172 cell line) [11]. More recently, Hohmann et al. observed that irradiation leads to changes in motility and to a less invasive phenotype of two glioblastoma cell lines, both associated with an increase of cell mechanical properties and changes in the cytoskeleton structure [12]. Our group reported similar results on healthy and tumor fibroblasts irradiated with 250 keV and 6 MeV [13,14,15]. A sensitive increase in the mechanical properties of tumor cells, responsible for the enhancement of cell-adhesion and the reduction of migration of tumor cells, was observed. The effects were significant and dose-dependent for the tumor cell line, while healthy fibroblasts resulted in being susceptible principally to high energy X-rays administered at doses higher than 1 Gy. Imaizumi et al. also observed response to X-irradiation strongly dependent on the dose administered to MDA-MB-231 breast cancer cells. If very low doses (0.5 Gy) enhanced cell migration and invasion, higher doses (2 and 10 Gy) suppressed MDA-MB-231 migration in a dose-dependent manner [16]. On the other hand, there are many studies reporting the opposite effect of ionizing radiation on cell motility. In particular, several works demonstrated that X-rays promote migration of neck and head carcinoma cells [17,18], breast cancer cells [19], central nervous system cells [19,20], lung cancer cells [21] and that this enhancement is sometimes accompanied by an increase of cell invasion [19,20], other times by a reduction [17]. Other in vivo experiments suggest that radiations can have a myriad of effects upon the motility and invasion capability of tumor cells. Numerous experimental and clinical studies have evidenced that in addition to a bystander effect that contributes to killing tumor cells, a non-targeted inhibitory effect on distant tumor growth (abscopal effects) exists, mediated by the response of the immune system [22]. However, other clinical trials support the idea that radiations contribute to a higher risk of metastasis through mechanisms mediated by the release of tumor cells into the circulation system or by effects into irradiated non-tumor cells [23]. The differences observed in these works highlight the complexity of the phenomenon of motility and/or invasion after irradiation and suggest wider investigations. To better understand the mechanisms underlying the effects of ionizing radiations on cell motility, here, we propose to consider the role of microenvironment, considering that properties, functions, and healthiness of the tissue are regulated by the close physical crosstalk existing between cells and ECM [24]. When these interactions are impaired, the mechanical integrity of tissue constituents (cells and ECM) changes, triggering cancer formation and progression [8]. Particularly for breast cancer, a large body of evidence has highlighted the existence of a close relationship between ECM stiffening, cancer cell softening and cancer onset, progression and aggression. In fact, on the one hand, breast cancer cells resulted in being significantly more deformable than the non-tumorigenic ones, and this change may facilitate easy migration and invasion of malignant cells during metastasis [25,26,27,28]. On the other hand, it has been widely demonstrated that the mechanics of the tumor microenvironment has a central role in the development of the disease. Stowers et al. showed that a protrusive and eventually invasive phenotype of non-transformed epithelial breast cells (MCF10A) can arise from the stiffening of the ECM matrix through the PI3K and Rac1 mechanotransduction pathway. In fact, the authors demonstrated that inhibition of these molecular pathways (Rac1, PI3K, MAPK, FAK, and ROCK) was able to suppress the invasive character of MCF10A cells [9]. Very recently, Panciera et al. observed that only a coordinated interplay between oncogene-mediated transformation and changes in the rigidity of the microenvironment is able to power up the process of tumorigenesis [9]. At the same time, the stiffening of ECM is also relevant to the progression of breast cancers. The increased cross-linking of collagen in breast cancer promotes focal adhesion formation, PI3K activity, as previously mentioned, and breast malignancy [24,29,30]. For this reason, the mechanical evaluation of breast tumor cells and ECM might provide new support to diagnosis and promote new insights in the field of chemo- and radiotherapy and clinical practice. In this work, we focus our attention on breast cancer, not only because it is one of the earliest models used to understand cancer progression and metastasis, but also because it is treated with RT from stages I (tumor dimension up to 2 cm and no lymph nodes involved) to III (tumor spread to lymph nodes or tissue near the breast) to reduce the risk of recurrence after surgery. In particular, we evaluated biophysical properties (strictly correlated to the cytoskeleton integrity, such as adhesion and migration) of two different mammary cell lines. A normal epithelial cell line (MCF10A) and a highly aggressive and invasive adenocarcinoma cell line (MDA-MB-231), on polyacrylamide (PAAm) substrates over a range of pathophysiological stiffness (1–13 kPa) in control conditions and after the exposure to two different doses of X-rays (photon beams), were investigated. Selected doses were 2 and 10 Gy, which represent the daily dose in radiotherapy treatment and the single maximum dose for the treatment of metastasis. Time points of 1 and 3 days (d) after irradiation were chosen based on our previous observations according to which, at these particular time points, healthy and tumor cells exhibited peculiar and different responses to irradiation [13,14,15]. Shedding light on the effects of X-rays on functions strictly associated with the cytoskeleton architecture and to cell-ECM crosstalk in healthy and tumor cells can open new frontiers in the diagnostic and therapeutic use of RT.

## 2. Results and Discussion

### 2.1. Role of Substrate Stiffness on Cell Morphological Features Before and after the Exposure to RT

To test the relevance of the mechanical properties of ECM on both healthy and tumor breast cell behavior and the response to the irradiation, we decided to modulate the mechanical properties of the cell culture substrate using PAAm hydrogels. Their mechanical properties were controlled by adding two different ratios of acrylamide and bis-acrylamide, and the rheological characterization verified that the elastic moduli of the two formulations were equal to 1.3 and 13 kPa, mimicking the values of stiffnesses of healthy breast tissue and malignant biopsies, respectively [29].

**Figure 1:**
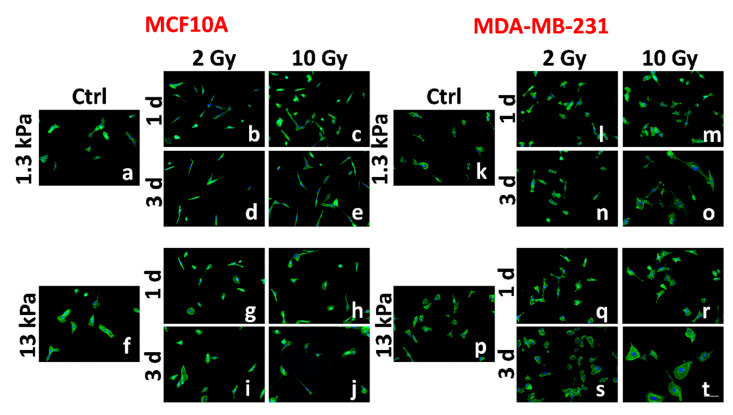
Representative images of two different mammary cell lines, MCF10A and MDA-MB-231, are shown. The spreading of MCF10A and MDA-MB-231 are compared before (**a**,**f**,**k**,**p**) and after RT (radiation therapy) (**b**–**e**,**g**–**j**,**l**–**o**,**q**–**t**). The cells were stained for F-actin (green) and nuclear DNA (blue). Scale bar, 100 μm.

**Figure 2:**
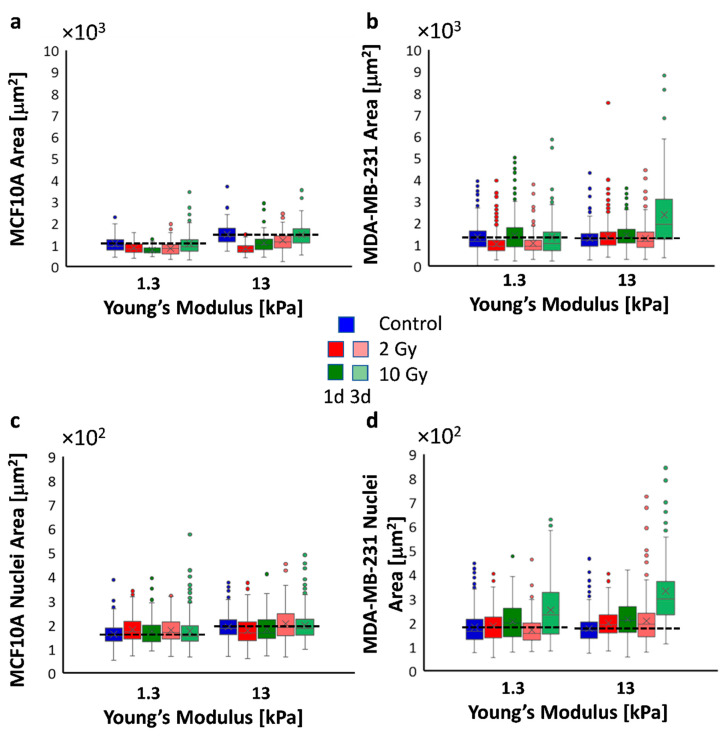
Box plots (mean, median, interquartile range, and outliers) of spreading areas (**a**,**b**) and nuclei areas (**c**,**d**). Spreading areas and nuclei area values were obtained from the analysis of Figure 1 and Figure S1). Dashed lines (**a**–**d**) indicate the mean values of spreading areas and nuclei areas in control conditions. *n* > 60 for cell spreading data, *n* > 110 for nuclear data.

It is now widely known that a strong relationship exists between the mechanical properties of the ECM and morphological features, such as cell spreading and nuclear shaping [31,32,33,34]. This relationship is strictly related to the myosin-tension generated inside the actin stress fibers necessary for their assembly and their associated focal adhesions, the structures deputed to the sensing of mechanical cues (also identified as mechanosensors). On the one hand, the actin cytoskeleton fosters the cell spreading in a proportional way to the tensional state generated inside it. The cytoskeleton, thanks to the LINC complex (Linker of Nucleoskeleton and Cytoskeleton), can transmit the mechanical forces to the nucleus, changing its shape, distorting the nuclear envelope, and evoking biochemical responses [32]. To evaluate the ability of MCF10A and MDA-MB-231 cells to sense and respond to the reactive forces originating by different mechanical properties of the substrate, we quantified their spreading, intended as the whole-cell area, and the nuclear area on collagen-functionalized PAAm substrates with different stiffness (1.3 and 13 kPa). Cells were plated on the substrates in sparse culture condition in order to avoid or limit the formation of adherens junctions that could impact on the adhesion and, more generally, on the mechanotransduction process [34]. We found that changes in substrate stiffness imposed different degrees of spreading and nuclear area in case of healthy cells, whereas, for metastatic cells, the same morphological features were not affected by the microenvironment stiffness at all (Figure 1a,f,k,p, Figure 2, and Figure S1; Table 1), suggesting compromised mechanosensing machinery. Furthermore, the spreading area of MDA-MB-231 on soft substrate resulted in being higher than that of MCF10A on the same substrate, in agreement with the increased formation and maturation of focal adhesions associated to enhanced intracellular contractility in KRAS-mutated cells cultured on 1 kPa substrate compared to MCF10A cells, as observed by Panciera et al. [10]. Panciera et al. also demonstrated that KRAS-mutated cells, like MDA-MB-231 cells, are characterized by a significant nuclear accumulation of YAP and TAZ, two well-established sensors of mechanotransduction, already when cultured on 1 kPa substrates. On the contrary, MCF10A cells exhibited a YAP/TAZ nuclear/cytoplasmic ratio lower than 1, indicating that YAP and TAZ are principally located inside the cytoplasmic compartment [10]. Consistently, we found higher values of the nuclear area of MDA-MB-231 on a soft substrate compared to MC10A cells.

Having characterized the mechanosensing activity of the two cell lines in the control condition, we evaluated the effects of irradiation on cell adhesion at 1 and 3 d after treatment at the two different doses of 2 and 10 Gy. At 1 d after irradiation, MCF10A cells resulted in being less spread compared to cells in the control condition. This effect was relevant for all conditions, even though more significant for cells cultured on a stiff substrate and irradiated with the lower dose (2 Gy; Figure 1a–c,f–h, Figure 2a, Figures S1 and S2; Table 1). Furthermore, the decrease of cell spreading resulted in being not dose-dependent on the soft substrate, whereas it exhibited an inverse dependence on the dose administered in case of cells cultured on the stiff substrate (Table 1). At the same time point, we found that the nuclear areas of irradiated healthy cells cultured on soft substrate increased slightly, but in a significant way and not dose-dependent (Figure 1a–c, Figure 2c, and Figure S1; Table 1). This was an unexpected result if associated with that of the spreading area. It could be explained supposing a protective mechanism, operated by microtubules and intermediate filaments on the nucleus and activated in a physiological environment. In contrast, on the stiff substrate, the nuclear area decreased in agreement with the spreading area, but without dependence on the dose administered (Table 1). At longer times, the effect on the spreading area was maintained only for the lower dose on both substrates, while the initial values were completely restored by the cells irradiated with the higher dose (10 Gy; Figure 1a–c, Figure 2a, and Figure S1; Table 1). The behavior of cells is more complicated if the data on the nuclear area are analyzed. In fact, the higher values were maintained by cells cultured on soft substrates and irradiated with lower dose indicating a more persistent effect of such dosage on cell adhesion, while the nuclear area of cells cultured on stiff substrates returned to its initial value (Figure 1f–j, Figure 2c, and Figure S1; Table 1).

The behavior of metastatic cells was significantly different. MDA-MB-231 cells resulted in being more sensitive to both doses of irradiation, even though the effects of the RT changed profoundly over time and with doses. In particular, at both time points, metastatic cells cultured on the physiological environment reduced their spreading area when irradiated with the dose of 2 Gy in a similar way to MCF10A cells (Figure 1l–n,q–s, Figure 2b, Figures S1 and S3; Table 1). On the other hand, their adhesion seemed not to be affected by the higher dose, indicating that, in this case, the microenvironment mimicking a healthy tissue mechanics (1.3 kPa) has a sort of protective role on cell properties (Figure 1m–o,r–t, Figure 2b, Figures S1 and S3; Table 1). Cells cultured on stiff substrates showed the opposite behavior. These cells, in fact, increased their spreading area significantly at 1 d in a dose-dependent manner. After 3 d, only cells irradiated with 2 Gy were able to regain their initial features, while for those cells treated with 10 Gy, the spreading area increased to a value 1.9-fold higher than in control condition (Figure 1d and Figure S1; Table 1). The effects of RT on nuclear areas of metastatic cells are reported in Figure 1k–t, Figure 2d, and Figure S1, and Table 1. In particular, the higher dose induced a significant increase of the nuclear area resulting 1.9-fold higher than in control condition (Figure 2d; Table 1), indicating that tumor cells, differently from the healthy ones, were more sensitive to 10 Gy than to 2 Gy dose. The results observed on stiff substrates are partially in agreement with those obtained by our group on MCF10A and MCF7 cells cultured on tissue culture plastics (3 GPa), which responded at the irradiation by, respectively, reducing and increasing their spreading area after irradiation [35]. The results point out the necessity not to neglect the role of mechanical microenvironment in regulating the response of cells to irradiation. The role of substrate stiffness and composition seems to take a regulatory effect on the response of metastatic cells to RT. In fact, Cordes et al. found that substrate, intended as ECM molecules (fibronectin and Matrigel), can have a radioprotective role on glioblastoma cells, which reflects in a significant upregulation of β-integrins by irradiation in its turn correlated with improved β-integrin-mediated adhesion to the substrate [8]. Our data, intersected with those previously obtained on petri-dish [35], indicate a range of stiffness, above 1.3 kPa and below 3 GPa, in which metastatic cells can recover the effect provoked by RT. It is well known, indeed, that ECM of tumoral tissues presents higher mechanical properties due to an increased percentage of aligned collagen fibers [8]. Hence, it is interesting that cells behave differently when cultured on substrates simulating stiffness higher than the physiological ECM of mammary epithelial cells.

Taken together, these results suggest how important is the role of the microenvironment and the necessity to perform further investigations to unravel new mechanisms underlying the response of healthy and tumor cells to RT treatments. In addition to this, observing the behavior of MCF10A and MDA-MB-231 cells after irradiation, it is important to recognize that, even if RT altered the biophysical properties of both cell lines, its impact was less relevant on healthy cells than on tumor cells. This suggests that MCF10A had a stronger ability to preserve or to recover their properties in both mechanical environments by adopting probable mechanisms of protection and repair. Furthermore, after RT tumor cells seem to restore a sort of mechanosensing process having again the ability to recognize the mechanical environment and to respond in a similar way to MCF10A cells.

### 2.2. Role of Substrate Stiffness on Cell Motility before and after the Exposure to RT

**Figure 3:**
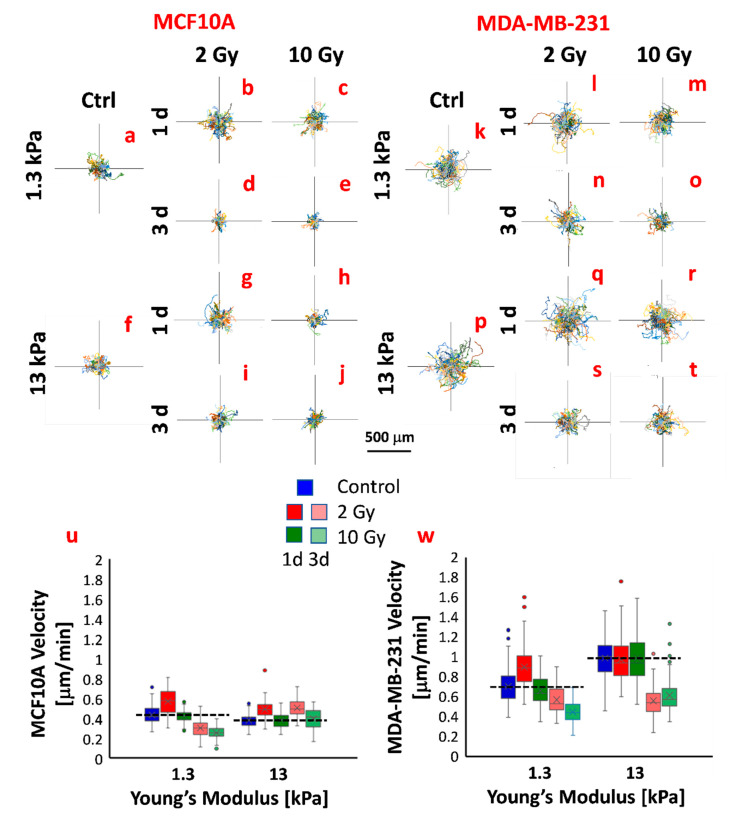
Plot at origin of trajectories of MCF10A and MDA-MB-231 before (**a**,**f**,**k**,**p**) and after RT (**b**–**e**,**g**–**j**,**l**–**o**,**q**–**t**). Trajectories of cells were obtained by manual tracking using ImageJ and Manual Tracking plugin (http://rsweb.nih.gov/ij/). Box plots (mean, median, interquartile range and outliers) of migration velocity of MCF10A (**u**) and MDA-MB-231 (**w**) in all analyzed conditions. Cell velocity was calculated by the trajectories using the formula 1 (see Section 3). Dashed lines (**u**,**w**) indicate the mean values of velocity in control conditions. *n* > 58 for all conditions.

**Figure 4:**
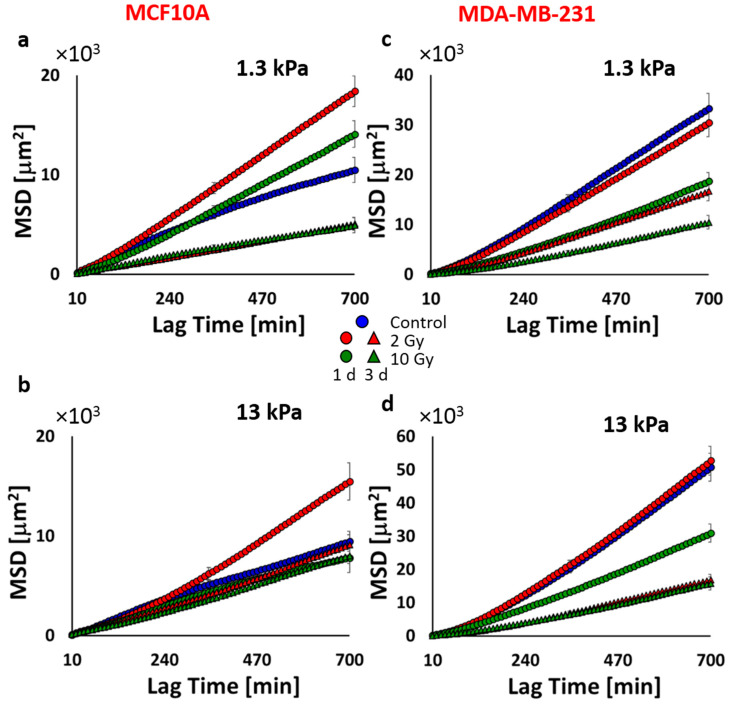
MSDs (mean square displacements) calculated from cell trajectories of MCF10A (**a**,**b**) and MDA-MB-231 (**c**,**d**) by using Formula (2) (see Section 3).

To evaluate MCF10A and MDA-MB-231 migratory behavior in response to substrate stiffness, cell videos were recorded with an interval of 10 min using time-lapse microscopy, and two essential parameters describing the efficiency of cell motility were computed: the mean migration velocity over a 24-h period and directional persistence. Whereas the migration velocity is easy to calculate and interpret, the persistence describes the time a cell employs to change its direction and has been estimated by fitting the mean square displacements (MSDs) over time with the Fürth’s formula (see Section 3). As shown in Figure 3u, the velocity of MCF10A decreased significantly from ~0.8 on the soft substrate to ~0.7 μm/min on the stiff substrate (Table 2), consistently with previous reports indicating that the migration velocity of healthy cells presents an inverse proportion to substrate stiffness [31]. This result can be explained by considering that cell motility is a complex process requiring repeated cycles of adhesion to and detachment from the ECM, strictly related to the focal adhesion life cycle (assembly–maturation–disassembly). In this regard, it has been widely demonstrated that when the stiffness increased, healthy cells form bigger stress fibers, contributing a more structured cytoskeleton, and longer focal adhesions with greater assembly/disassembly rate that slows down the cell migration velocity [36]. Moreover, the persistence time resulted in being equal to 0 (Figure 4a,b; Table 3), indicating that the motility of MCF10A cells reflects the behavior of a random Brownian motion on both substrates. On the contrary, the motility of MDA-MB-231 cells increased with the increase of substrate stiffness passing from ~0.7 to ~1 μm/min (Figure 3v, Table 2), consistently with other studies reporting the same effect on the migration of various cancer cells, including pancreatic cancer cells, colorectal cancer cells, breast cancer cells, and so forth [37,38,39,40,41]. What emerged from our experimental results was that MDA-MB-231 cells were more directionally stable on both substrate stiffness compared to healthy cells and also their persistence time was positively correlated to the substrates stiffness, resulting close to 1 and 2 h on soft and stiff substrates, respectively (Figure 4c,d; Table 3). This finding indicated that the ECM stiffening promotes not only motility but also the ability of tumor cells to invade distant sites by increasing the directional persistence, in agreement with observations made in vitro and in vivo experiments. In particular, Levental et al. clearly showed that in breast tumors, the crosslinking of collagen and ECM stiffness regulate the invasive behavior of oncogene pre transformed epithelial cells (H-RAS-transformed MCF10A expressing high levels of activated H-RAS in a similar way to MDA-MB-231 cells) [29]. In addition, the mean migration velocity of MDA-MB-231 cells on the stiffer substrate is significantly higher than that of MCF10A cells, indicating that in the absence of pre-oncogene activity, ECM stiffening does not drive mammary epithelial cell metastasis [10,29]. These two observations, the direct relationship between cell migration velocity and ECM stiffness and the higher velocity of tumor cells compared to that of healthy cells, can be explained by considering that tumor cells have a less organized cytoskeleton with lower mechanical properties, fewer and less-developed focal adhesion than their normal counterpart [42,43]. At the same time, malignant cells exhibit a very high level of focal adhesion kinase (FAK) [44], a nonreceptor tyrosine kinase activated by integrin clustering and involved in the process of disassembly of focal adhesion, and the overexpression and activation of FAK are promoted by the stiffening of the ECM [45]. Consequently, the upregulation of FAK on stiff ECM contributes to increasing the rate of assembly/disassembly of focal adhesions without allowing their maturation and the formation of a robust cytoskeleton but promoting tumor cell invasion (directional persistence) and migration (migration velocity) instead [44].

Cell motility analysis showed that irradiation had important effects also on the migratory behavior of healthy and tumor cells. At 1 d after irradiation, MCF10A cells cultured on both physiological and supraphysiological stiffnesses and irradiated with the lower dose responded by increasing their migration velocity (Figure 3u, Table 2), as a consequence of their reduced adhesion (Figure 2a), while the high dose did not seem to affect their motility, even though in this case the adhesion also presented lower values (Figure 2a). As demonstrated by the increase of the persistence time and compared to the control conditions, the motility mode changed sensitively for those conditions in which cell velocity increased (cells irradiated with 2 Gy and 10 Gy and cultured on the stiff substrate; Figure 4a,b; Table 3). At a longer time, cells cultured on soft substrate reduced their velocity in a very significant way, showing a partial concordance with RT effects on the adhesion (Figure 3u, Table 2). In fact, whereas cells irradiated with the higher dose increased their adhesion to the substrate, those irradiated with the lower dose continued to exhibit a reduced adhesion (Figure 2a). At 3 d after irradiation, the persistence of 2 Gy-irradiated cells was lower than those observed at 1 d but higher than those of control cells. This indicates that irradiation can have a strong impact on cell adhesion and motility, both in terms of velocity and directionality (Figure 4a,b; Table 3). According to the results on cell adhesion, the low dose produced effects more durable than the high dose. This, in particular on the stiff substrate, produced a reduction of the spreading area and an enhancement of migration velocity and directional persistence.

MDA-MB-231 cells responded to RT by changing their velocity and persistence time, but the latter remaining in all cases higher than 1 h and retaining the natural propensity of these cells to move directionally. MDA-MB-231 cells on soft substrate responded to RT by increasing their migration velocity when irradiated with 2 Gy, but this effect was completely overturned at 3 d, inducing cells to move slower (Figure 3w, Table 2) and more persistently as a consequence of the reduced adhesion (Figure 4c,d; Table 3). The dose of 10 Gy on cells on the soft substrate produced a time-dependent reduction of migration velocity and, also, in this case, an increase of directional persistence (Figure 3w, Table 2 and Table 3). On the other hand, MDA-MB-231 cells cultured on stiff substrate had a very similar response to both irradiation; in fact, their velocity remained unchanged 1 d after irradiation, but the persistence was unperturbed for the low dose and reduced for the high one. At 3 d after irradiation and irrespective of the dose delivered, we measured a significantly reduced migration velocity as a possible consequence of the increased adhesion (Figure 2b). To summarize, the above findings suggest that at 3 d after irradiation, tumor cells exhibited reduced migration, even if their persistence was not substantially changed by dose delivered. This effect was particularly relevant for tumor cells on ECM mimicking tumor microenvironment, which decreased their velocity from ~1 to 0.6 μm/min, approaching the velocity value of healthy cells on stiff ECM (~0.4 μm/min). What appears very clear is the importance of the microenvironment in mediating the cellular response to a physical insult such as photon irradiation. To better elucidate the mechanisms underlying the effect of RT on cell functions in relationship to the mechanics of ECM, it will be necessary to evaluate the effects of radiations on the expression of molecules involved in both processes, first of all, integrins and FAK. In fact, it has been shown that both integrins and FAK regulate biological processes necessary for the pathogenesis of cancer. Paszek et al. suggested that tumor stiffness contributes to the aberrant behavior of epithelial tissue by modulating integrin signaling through FAK [45]. In addition, irradiation can affect the expression of integrins and have serious repercussions on adhesion and invasion. As previously mentioned, Cordes et al. found that ionizing radiation promoted integrin expression in a substrate-dependent way, improving cell adhesion on fibronectin and Matrigel and impairing their invasion ability [11]. Oppositely, Rieken et al. indicated that the overexpression of integrins induced by photons had a promigratory effect on the same tumor cells [46]. On the other side, it will be fundamental also to explore the effects of irradiation on the architecture and mechanical properties of ECM. Our findings, indeed, indicate the importance of including its contribution and crosstalk with cell cytoskeleton architecture in the attempt to reconstruct all the pieces when deciphering cell response to RT treatments.

## 3. Materials and Methods

### 3.1. Preparation of Substrate and Mechanical Characterization

Polyacrylamide substrates were prepared as previously reported [47], with minor modifications. Briefly, glass-bottom culture dishes (World Precision Instruments, FD35−100) were silanized with 3-aminopropyltriethoxysilane (Sigma-Aldrich, St. Louis, MO, USA) for 20 min and extensively washed with water. Then, 40% acrylamide and 2% methylene-bis-acrylamide were mixed in phosphate-buffered saline (PBS) solution at two different final concentrations: 4% acrylamide/0.15% methylene-bis-acrylamide and 10% acrylamide/0.1% methylene-bis-acrylamide corresponding to 1.3 and 13 kPa (Young’s modulus), respectively. Polymerization was initiated by adding 1/100 total volume of 10% ammonium persulfate and 1/1000 total volume N,N,N′,N′-tetramethylethylenediamide (TEMED, Sigma-Aldrich, T7024, St. Louis, MO, USA). Ten microliters of acrylamide/methylenebis-acrylamide mixture were pipetted on the treated dishes and covered with a 10-mm coverslip. After 20 min, the coverslip was removed and PBS was added to the dish. Before functionalization, the substrates were soaked with a penicillin–streptomycin solution overnight and then exposed to UV light emitted by a germicidal lamp for 1 h. After sterilizations, substrates were functionalized with collagen by using a bifunctional photoreactive crosslinker (sulfosuccinimidyl 6-(4’-azido-2’-nitrophenylamino) hexanoate, sulfo-SANPAH; Fischer Scientific, Loughborough, UK). The sulfo-SANPAH solution was diluted in water at a final concentration of 0.2 mg/mL, placed on PAAm substrates, and exposed to 365 nm UV for 10 min. After washing with PBS, the substrates were incubated with a solution of bovine type I collagen at the final concentration of 50 μg/mL for 2 h at 37 °C. Finally, samples were washed with PBS.

After the process of photoactivation, the sulfo-SANPAH solution was removed, and 0.25 mL solution of PBS and 2% bovine collagen was added to every dish before being incubated at 37 °C for 2 h.

### 3.2. Cell Culture

The MDA-MB-231 cell line was cultured in Lonza Dulbecco’s modified Eagle medium (DMEM/F-12) supplemented with 10% fetal bovine serum (FBS, Gibco, Eggenstein, Germany), 1% L-glutamine (Sigma, St. Louis, MO, USA) and 1% penicillin–streptomycin (Sigma, St. Louis, MO, USA).

The MCF10A cell line was cultured in Lonza mammary epithelium-based medium (MEBM), supplemented with the Mammary Epithelial Cell Growth Medium SingleQuots Kit (MEGM): bovine pituitary extract (BPE), human epidermal growth factor (hEGF) (0.1%), insulin (0.1%), hydrocortisone (0.1%), gentamicin–amphotericin (GA-1000; 0.1%).

### 3.3. Cell Irradiation

MCF10A and MDA-MB-231 cell lines were exposed to X-rays (photon beams) delivered by the LINAC Synergy Agility (ELEKTA), using a 6MeV energy beam, at the National Cancer Institute “PASCALE” of Naples. Three-dimensional conformal radiation therapy (3D-CRT) treatment plans were realized with Monaco v5.11.03 TPS (treatment planning station) by Elekta to deliver 2 and 10 Gy, with the following setup. Cells were settled in a Petri dish between two solid water phantom slabs (2 and 3 cm each) and exposed at two opposite fields. The dose rate selected was 200 UM/min. Prescribed doses were delivered on a uniform square field of 20 × 20 cm^2^ at the cell level.

The 3D-CRT approach was chosen for the simulation, being broadly used in clinical practice, although breast cancer treatment can be performed by several other radiotherapy techniques, as IMRT (intensity-modulated radiation therapy) and VMAT (volumetric-modulated arc therapy).

The 2 Gy represents the conventional fractional dose delivered in standard treatment, whereas 10 Gy was selected to represent the highest dose/fraction treatment as stereotactic body radiotherapy, even though rarely employed for breast cancer.

### 3.4. Cell Adhesion Analysis

MCF10A and MDA-MB-231 cells were cultured on PAAm substrates at a final density of 1000 cells/cm^2^. Cells were fixed and immunostained for cell spreading and nuclear area in the control condition and 1 and 3 d after irradiation. Cells were washed with PBS and fixed in 4% paraformaldehyde for 20 min, rinsed twice with PBS, permeabilized with 0.1% Triton X100 for 5 min, and blocked with 10% goat serum for 1h. Cells were incubated with Alexa 488 phalloidin at 1:200 dilution. Finally, nuclei were stained with Hoechst 33342. Images of cells were acquired using an Olympus IX81 inverted microscope and a 10× objective. Images were imported into ImageJ software (NIH, Bethesda, MD, USA) for quantification of cell spreading and nuclei area. Individual cells and nuclei were thresholded manually on the basis of phalloidin and nuclei staining, and their spreading and nuclei areas were determined using the “Measure” command in ImageJ.

### 3.5. Cell Motility

MCF10A and MDA-MB-231 cells were cultured on PAAm substrates at a final density of 1000 cells/cm^2^. Cell motility experiments were performed by time-lapse microscopy (Olympus IX81 with 4×). Phase-contrast images were acquired at 10 min interval for 24 h for a total number of 144 frames. Cell positions in each frame were tracked manually using ImageJ and Manual Tracking plugin (http://rsweb.nih.gov/ij/). Migration velocity and MSD were calculated, starting from trajectories using the following formula:

(1) $$v=\sum \frac{\sqrt{{\left(x_{i+1}-x_{i}\right)}^{2}+{\left(y_{i+1}-y_{i}\right)}^{2}}}{\Delta T}$$

(2) $$MSD\left(\tau \right)={\left[x\left(t-\tau \right)-x\left(t\right)\right]}^{2}+{\left[y\left(t-\tau \right)-y\left(t\right)\right]}^{2}$$

where *x_i_* *e* *y_i_* were the coordinates of cell in the *i*-th frame, Δ*T* is the time interval between two frames, *t* is the time, and *τ* is the lag time.

To estimate diffusion coefficient D and directional persistence P, MSDs curves were fitted with Fürth’s Formula [48]:

(3) $$MSD\left(\tau \right)=4D\left(\tau -P\left(1-e^{-\frac{\tau }{P}}\right)\right)$$

The fitting was done with ordinary nonlinear least-squares regression analysis.

### 3.6. Statistical Analysis

Statistical comparisons were performed with a Student’s unpaired test when data exhibit a normal distribution. Otherwise, a nonparametric Mann-Whitney test was used. *P*-values of <0.05 denote statistically significant differences.

## 4. Conclusions

In summary, our findings indicate that ECM mechanics can play a very active role in mediating responses of cells to RT. In particular, RT had significant effects on biophysical properties of MDA-MB-231 cells cultured on stiff ECM mimicking tumor environment, whereas their adhesion and nuclei resulted in being less affected when cells interact with substrate mimicking a physiological mechanical environment. MCF10A features were less affected by RT on both substrates, suggesting a stronger ability of these cells to preserve themselves, at least in terms of spreading and nuclei morphology. On the contrary, the migration velocity of both cell lines was significantly reduced on soft substrate, indicating a sort of radioprotective role of physiological ECM that impaired cell motility and invasion. These preliminary findings, together with further and thorough examinations, can shed light on the ECM-mediated molecular mechanism underlying the response of healthy and tumor cells to radiation treatments, generate data with a higher translational significance, and pave the way for exploring new frontiers in the diagnostic and therapeutic use of RT.

## Figures and Tables

**Table 1:** Statistical analysis for data of spreading and nuclei area.

			Control	2 Gy				10 Gy			
				1 day		3 days		1 day		3 days	
			13 kPa	1.3 kPa	13 kPa	1.3 kPa	13 kPa	1.3 kPa	13 kPa	1.3 kPa	13 kPa
**Control**		1.3 kPa	***.NS ***.NS	**.### ***.NS	***.NS **.###	***.### ***.NS	NS.NS ***.###	**.NS NS.##	***.NS NS.###	NS.NS NS.###	***.### ***.###
		13 kPa		***.### **.#	***.# ***.###	***.### **.NS	***.NS NS.###	***.## ***.###	***.### **.###	***.NS ***.###	NS.### NS.###
**2 Gy**	**1 day**	1.3 kPa			NS.### NS.#	*.NS NS.###	***.### ***.NS	NS.### NS.#	***.### NS.###	*.## **.###	***.### ***.###
		13 kPa				NS.### NS.###	***.NS ***.NS	NS.NS NS.NS	***.NS NS.NS	***.## NS.###	***.### ***.###
	**3 days**	1.3 kPa					***.### ***.###	NS.### NS.###	***.### NS.###	***.NS *.###	***.### ***.###
		13 kPa						***.# ***.NS	***.NS **.NS	NS.NS ***.###	***.### **.###
**10 Gy**	**1 day**	1.3 kPa							***.## NS.NS	*.## NS.##	***.### ***.###
		13 kPa								***.NS *.NS	***.### **.###
	**3 days**	1.3 kPa									***.### ***.###

Asterisks (*) refer to spreading area (blue) and nuclei area (red) of MCF10A cell, hash signs (#) to those of MDA-MB-231 cells. ***, ### *p* < 0.001, **, ## *p* < 0.01, *, # *p* < 0.05; NS—not significant.

**Table 2:** Statistical analysis for motility data.

			Control	2 Gy				10 Gy			
				1 day		3 days		1 day		3 days	
			13 kPa	1.3 kPa	13 kPa	1.3 kPa	13 kPa	1.3 kPa	13 kPa	1.3 kPa	13 kPa
**Control**		1.3 kPa	***.###	***.###	***.###	***.###	***.###	**NS.NS**	***.###	***.###	***.###
		13 kPa		***.##	***.NS	***.###	***.###	***.###	**NS.NS**	***.###	***.###
**2 Gy**	**1 day**	1.3 kPa			***.#	***.###	***.###	***.###	***.#	***.###	***.###
		13 kPa				***.###	**NS.###**	***.###	***.NS	***.###	***.###
	**3 days**	1.3 kPa					***.NS	***.###	***.###	***.###	***.NS
		13 kPa						***.###	***.###	***.###	***.NS
**10 Gy**	**1 day**	1.3 kPa							**.###	***.###	***.###
		13 kPa								***.###	***.###
	**3 days**	1.3 kPa									***.###

Asterisks (*) refer to cell velocity of MCF10A cells, hash signs (#) to those of MDA-MB-231 cells. ***, ### *p* < 0.001, **, ## *p* < 0.01, # *p* < 0.05; NS—not significant.

**Table 3:** Parameters describing the motility of cells.

		Control		2 Gy				10 Gy			
				1 d		3 d		1 d		3 d	
		1.3 kPa	13 kPa	1.3 kPa	13 kPa	1.3 kPa	13 kPa	1.3 kPa	13 kPa	1.3 kPa	13 kPa
**MCF10A**	**D [mm^2^/min]**	4.0448	3.4916	6.9223	7.8180	1.8238	3.3666	5.4219	2.9704	1.8234	3.0483
	**P [min]**	0.0090	0.0086	32.2139	113.1808	6.6790	31.9583	49.0114	0.05537	0.03201	43.5825
	**R^2^**	0.9864	0.9937	0.9997	0.9987	0.9993	0.9973	0.9996	0.9939	0.9943	0.9979
**MDA-MB-231**	**D [mm^2^/min]**	13.0096	21.4693	11.8329	22.1579	6.7391	7.305239	7.594923	12.08702	4.338288	6.487246
	**P [min]**	59.7990	114.9188	62.1477	112.1086	84.9445	122.0365	97.8991	71.3609	104.0150	100.9757
	**R^2^**	0.9998	0.9995	0.9998	0.9995	0.9998	0.999801	0.998634	0.998922	0.999376	0.998346

Values of diffusion coefficient (D), directional persistence (P), and goodness-of-fit (R^2^) obtained by fitting the MSD (mean square displacements) of cells’ trajectories to Fürth’s formula.

## Supplementary Materials

The following are available online at https://www.mdpi.com/2072-6694/12/5/1170/s1, Figure S1: Zoomed pictures of spreading of MCF10A and MDA-MB-231 cells, Figure S2: Percentage distribution of cell spreading area of MCF10A cells before and after RT, Figure S3: Percentage distribution of cell spreading area of MDA-MB-231 cells before and after RT.
