# Supplementary material for: Adhesion and Migration Response to Radiation Therapy of Mammary Epithelial and Adenocarcinoma Cells Interacting with Different Stiffness Substrates
Source: Cancers (Basel). 2020 May 6;12(5):1170. doi: 10.3390/cancers12051170 (PMC7281676; doi:10.3390/cancers12051170)
Supplement: Supplementary file 1 [file cancers-12-01170-s001.pdf]

cancers

Adhesion and Migration Response to Radiation Therapy of Mammary Epithelial and Adenocarcinoma Cells Interacting with Different Stiffness Substrates

Supplementary Information

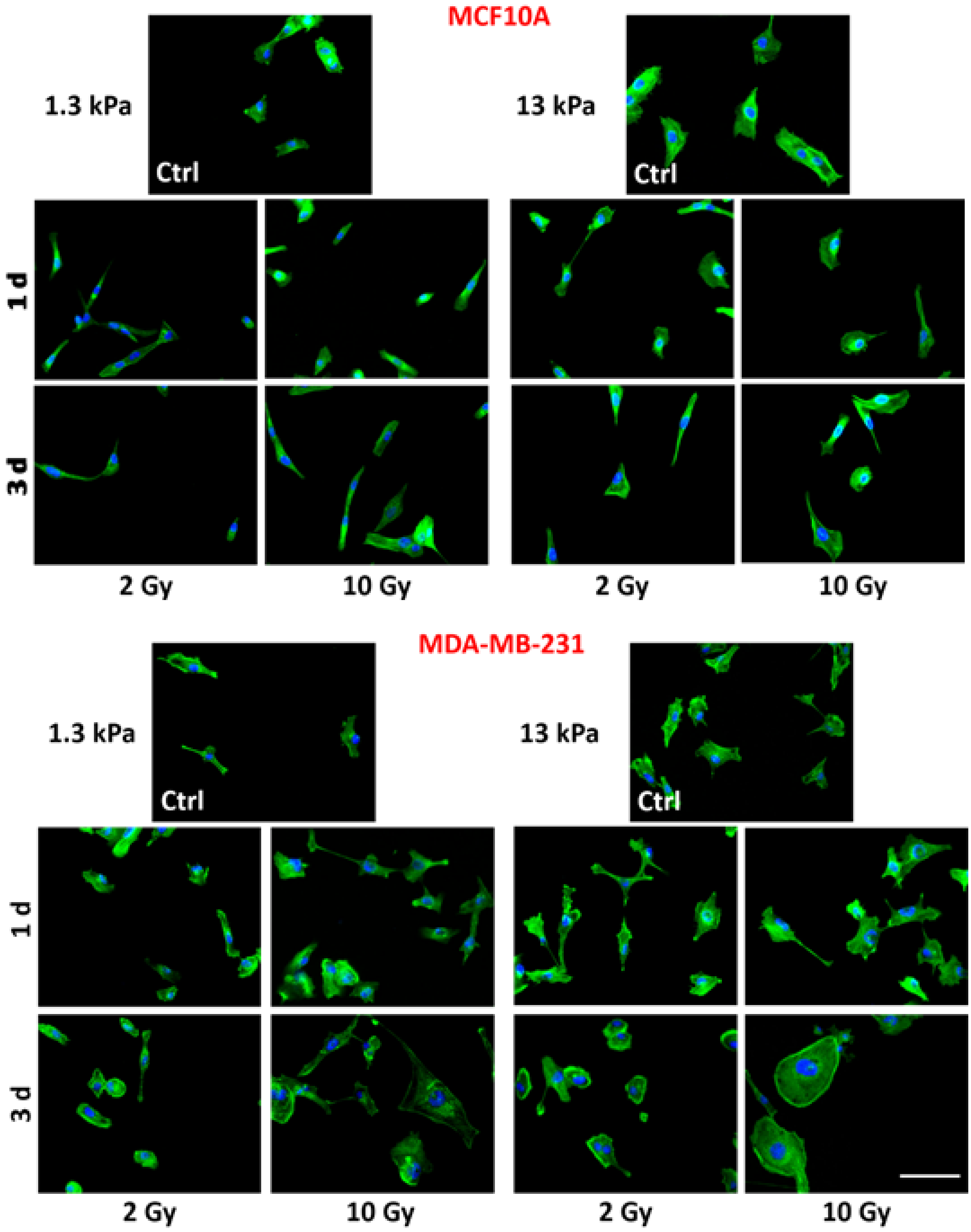

Figure S1. Zoomed pictures of spreading of MCF10A and MDA-MB-231 are compared before and after RT (radiation therapy). The cells were stained for F-actin (green) and nuclear DNA (blue). Scale bar, 100 μm.

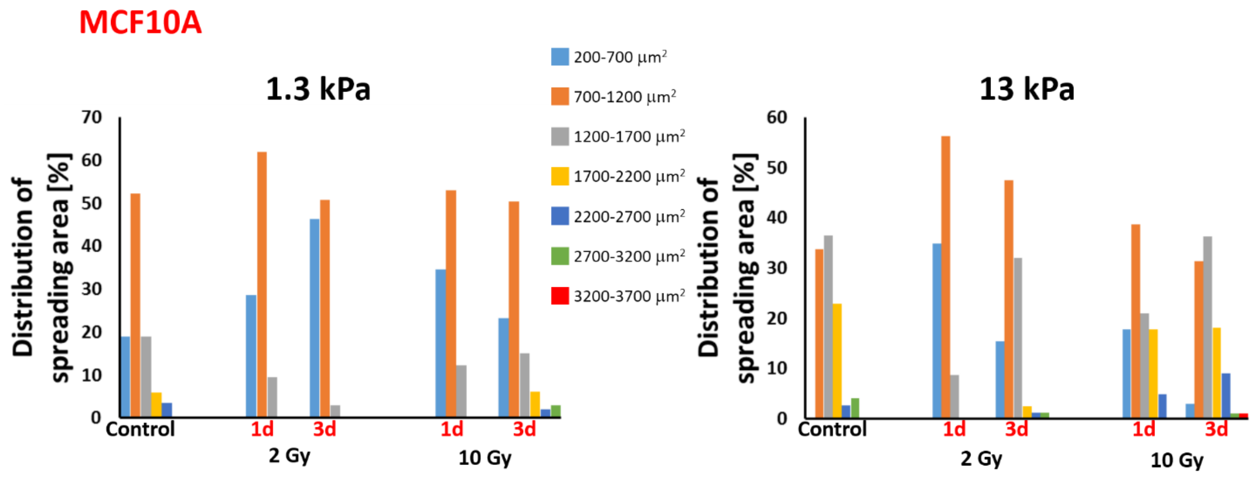

Figure S2. Percentage distribution of cell spreading area of MCF10A cells before and after RT on soft (1.3 kPa) and stiff (13 kPa) substrates.

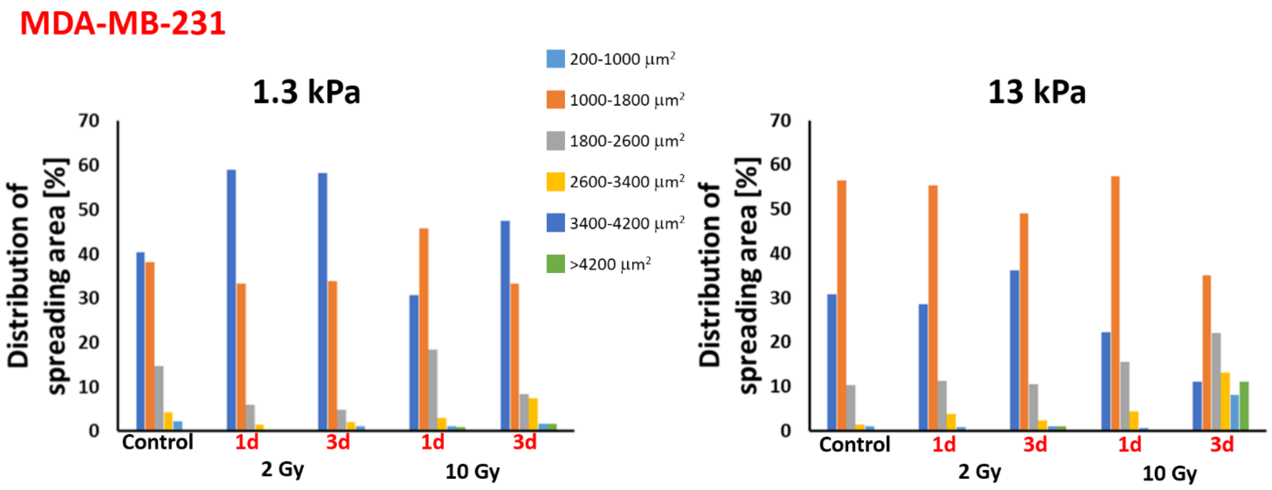

Figure S3. Percentage distribution of cell spreading area of MDA-MB-231 cells before and after RT on soft (1.3 kPa) and stiff (13 kPa) substrates.
